# Adherence to a Gluten Free Diet Is Associated with Receiving Gluten Free Foods on Prescription and Understanding Food Labelling

**DOI:** 10.3390/nu9070705

**Published:** 2017-07-06

**Authors:** Humayun Muhammad, Sue Reeves, Sauid Ishaq, John Mayberry, Yvonne M. Jeanes

**Affiliations:** 1 Health Sciences Research Centre, University of Roehampton, London SW15 4JD, UK; muhammah@roehampton.ac.uk (H.M.); s.reeves@roehampton.ac.uk (S.R.); 2 Gastroenterology, Dudley Group NHS Foundation Trust, Dudley DY1 2HQ, UK; Sauid.Ishaq@dgh.nhs.uk; 3 Gastroenterology, University Hospitals of Leicester, Leicester LE1 5WW, UK; John.mayberry@uhl-tr.nhs.uk

**Keywords:** coeliac disease, gluten free, dietary adherence, prescription

## Abstract

Treatment of coeliac disease requires a strict gluten-free (GF) diet, however, a high proportion of patients do not adhere to a GF diet. The study explores the practical challenges of a GF diet and dietary adherence in Caucasian and South Asian adults with coeliac disease. Patients with biopsy- and serology-proven coeliac disease were recruited from a hospital database. Participants completed a postal survey (*n* = 375), including a validated questionnaire designed to measure GF dietary adherence. Half of Caucasians (53%) and South Asians (53%) were adhering to a GF diet. The quarter of patients (*n* = 97) not receiving GF foods on prescription had a lower GF dietary adherence score compared with those receiving GF foods on prescription (12.5 versus 16.0; *p* < 0.001). Not understanding food labelling and non-membership of Coeliac UK were also associated with lower GF dietary adherence scores. A higher proportion of South Asian patients, compared with Caucasians, reported difficulties understanding what they can eat (76% versus 5%; *p* < 0.001) and understanding of food labels (53% versus 4%; *p* < 0.001). We recommend retaining GF foods on prescription, membership of a coeliac society, and regular consultations with a dietitian to enable better understanding of food labels. Robust studies are urgently needed to evaluate the impact of reducing the amount of GF foods prescribed on adherence to a GF diet in all population groups.

## 1. Introduction

Coeliac disease (CD) is a common problem and affects 1% of the population [1]. Lifelong exclusion of gluten from the diet is the only practical and successful treatment available for people with CD [2]. A gluten-free (GF) diet can be considered restrictive because it excludes many commonly consumed carbohydrate based foods. A GF diet usually consists of a combination of naturally GF foods (e.g., potatoes, rice, unprocessed meat, fruit, and vegetables), plus manufactured GF versions of wheat-based foods, such as bread, cereals, and pasta. Adhering to a GF diet can be very challenging, it requires knowledge, skills, and modified behaviours to undertake substantial changes to dietary habits, including within social situations [3,4]. There are additional issues regarding the availability and cost of manufactured GF versions of wheat-based foods. Adherence to a GF diet ranges from 36% to 96% depending on the study method used to determine dietary adherence and is associated with a variety of demographic, psychosocial, and clinical factors [5,6,7,8,9,10]. Gluten ingestion results in villous damage and subsequently increases the risk of anaemia, osteoporosis, and malignancy for patients with CD [11,12]. Our study focuses on the practicalities of following a GF diet, including access to GF foods. 

The availability and cost of manufactured GF versions of wheat-based foods have been linked to dietary adherence [6,9,13]. In England, access to manufactured GF versions of wheat-based foods on prescription is changing. Prescribing guidelines recommend 18 units per month for adults [14] where 1 unit = 400 g loaf of bread. However, there is disparity across England as to the number of units prescribed and whether GF foods can be obtained on prescription at all. Local clinical commissioning groups (CCGs), of which there are 207 in England, decide who can access GF foods on prescription. Approximately 40% of CCGs are currently restricting or removing gluten-free food prescriptions in England [14]. In recent years there has been an improved availability of manufactured GF versions of wheat-based foods in regular and quality supermarkets, as well as online, but they remain significantly more expensive than gluten containing alternatives [15,16]. Budget supermarkets and local ‘corner’ shops which tend to be frequented by patients from lower socioeconomic groups often stock no GF alternatives [15,16].

Even when patients are receiving GF foods on prescription, Violato and Gray [17], reported a mean increase of £861 per year on food shopping after diagnosis with CD. The removal of prescriptions for GF foods could lead to financial hardship [18] and a situation similar to that in the U.S., whereby the perceived cost of GF foods is significantly associated with reduced adherence to a GF diet [9]. The current trend for CCGs restricting the prescription of manufactured GF versions of wheat-based foods [19], coupled with the high costs and limited availability of GF foods (compared with gluten containing foods) leads to real concerns over long-term health risks for people with CD. Our study aims to explore if practical factors have an impact on adherence to a GF diet in adult Caucasians and South Asians with CD residing in England.

There is a paucity of information regarding GF dietary adherence in South Asians with CD. Over 10 years ago a small cohort study of South Asians residing in England (*n* = 21) reported that they were dissatisfied with the health services received and a low proportion joined a coeliac society, however, there were no factors reported that were significantly linked to GF dietary adherence [20]. 

## 2. Methods

This cross-sectional study identified 972 patients from a hospital database with histologically-confirmed CD with supporting positive serology, (either tissue transglutaminase or endomysial antibodies) from which patients diagnosed between 2004 and 2014 were selected. The study location has a rich ethnic mix, Asian or Asian British residents are the largest ethnic minority group; thus, the researchers aimed to capture data on this population group. A postal invitation was sent to all patients (>18 years old) in 2015 offering support in seven ethnic languages. Once informed consent was received demographic data was collected from the CD database. Patients completed a previously-published questionnaire collecting information on diagnosis, dietary habits, symptoms, and interaction with both physician and dietitian clinics and the practical difficulties faced when following a GF diet [21]. Though patients were not involved in the design of the study, patient responses from other published studies were taken into consideration. The validated Coeliac Dietary Adherence Test (CDAT) was selected to indicate adherence to a GF diet [22], it consists of seven items on a Likert scale, the sum of the numeric values provide a score ranging from five to 35; lower scores reflect better adherence to a GF diet. A score of 13 or less was previously determined to accurately predict an adequate adherence taking the assessment of an expert dietitian as the gold standard [9]. During the time of data collection, national guidance of 18 units of prescribed GF food was in place for the area of recruitment, GPs had the ultimate decision on the amount of GF foods to prescribe and data was not collected on actual units prescribed to patients.

Data was analysed using SPSS statistical package version 22 (IBM Corp. Armonk, NY). Data was assessed for normality and Mann–Whitney was used to compare CDAT scores. CDAT scores are presented as medians with interquartile range (IQR). Spearman’s correlation between age and CDAT score, and Chi-squared for categorical variables. Logistic regression with predictor values entered simultaneously was used to explore variance of adherence to a GF diet. All patients gave their informed consent for inclusion before they participated in the study. The study was conducted in accordance with the Declaration of Helsinki, and the protocol was approved by the procedures of the University of Roehampton and Health Research Authority approval REC number 14/LO/2128.

## 3. Results

### 3.1. Response Rates and Diagnosis

The questionnaires were completed by 375 adults with coeliac disease; a return rate of 39%. Those who completed the questionnaire were significantly younger than those who did not respond (48 ± 18 versus 51 ± 18 years; *p* = 0.005), male and female responses were not significantly different. A significantly lower proportion of South Asians returned the questionnaire compared with Caucasians (26.6% versus 40.7%; χ^2^, phi −0.10, *p* = 0.001). There were 267 females and 108 males in our cohort; 337 Caucasians and 38 South Asians. The mean age was 48 years (SD 17.6). Peak diagnosis was between age 18 and 30 years (*n* = 100) with a female predominance of 79%, and a second peak in diagnosis was observed between 51 and 60 years (*n* = 84) with male dominance (61%).

During their consultation with a consultant, at the time of their diagnosis, 89% recalled they had been informed of the need to follow a strict GF diet, with 92% receiving a referral to a dietitian. The majority reported satisfaction with the information received from their consultant (South Asians 97%, Caucasians 95%; *p* = 0.51). Of those referred to a dietitian, 94% recalled the dietitian explained the diagnosis and reasons for a GF diet, 97% received an information pack, 89% recalled a follow up appointment, and 86% had a contact telephone number. Overall, 93% were satisfied with the information received from the dietitian (South Asians 100%, Caucasians 91%; *p* = 0.055) and 88% agreed the dietitian should play an important role in the long-term management of CD.

### 3.2. Gluten-Free Dietary Adherence

The majority of patients self-reported never knowingly ingesting gluten (62%), however, only 52% adhered to the GF diet when assessed by the GF dietary adherence CDAT score (> 13 non-adherent) (χ^2^, phi 0.70, *p* < 0.001). Similarities in CDAT scores were observed between all South Asians and Caucasians, however, there was a significantly higher CDAT score for South Asian women compared with Caucasian women (Table 1). There was a weak negative correlation between age and CDAT score (*r* = −0.11; *p* = 0.03), indicating older patients may adhere better to a GF diet. CDAT scores were similar for patients who were diagnosed before 18 years (*n* = 34) with those diagnosed in adulthood (*n* = 341; *p* = 0.49). From the 139 patients, who self-reported ingesting gluten at least once a month, only 84 reported symptoms after gluten ingestion (symptoms with gluten ingestion CDAT score 20, no symptoms CDAT score 19; *p* = 0.72). 

### 3.3. Practical Factors Influencing GF Dietary Adherence

Not understanding food labels was significantly associated with poorer GF dietary adherence CDAT score (Table 2); 73% of those who reported not understanding food labels were classified as not adhering to a GF diet compared with 45% who understood food labels (χ^2^, phi −0.16; *p* = 0.02). Over half of South Asians reported difficulty in understanding food labels compared with just 4% of Caucasians (53% versus 4%; χ^2^, phi −0.52, *p* < 0.001, Table 3). South Asians who did not understand food labelling (*n* = 20) had a significantly poorer GF dietary adherence CDAT score (19 (13–24)) compared with South Asians who understood food labelling (12 (9–13.5), *n* = 18; *p* < 0.001). 

Of the patients not receiving GF foods on prescription (*n* = 97), 62% were classified as not adhering to a GF diet (CDAT score >13), compared with 42% of those receiving GF foods on prescription (χ^2^, phi 0.17, *p* = 0.001). Significantly lower CDAT scores are reported for patients who received GF foods on prescription, compared with those who did not (12.5 verses 16; *p* < 0.001, Table 2). A higher proportion of South Asians were receiving GF foods on prescription than Caucasians (Table 3). Of the 278 (74%) patients who received GF free products on prescription 75% reported receiving sufficient GF products on prescription; at the time of data collection the national guidance of 18 units per month was in place. The majority of people with CD (*n* = 301, 80%) stated that GF products were expensive (including those receiving GF prescriptions (80%) and those not (81%:NS)).

The majority of patients responded that they had received information about Coeliac UK in their consultation with a dietitian (*n* = 352, 93%), though only 202 of those patients (53%) joined Coeliac UK. Patients who were members of Coeliac UK had a significantly lower CDAT score, compared with non-members (12 verses 14.5; *p* < 0.001, Table 2). The most common reason cited for not being a member was ‘they did not feel it was important to be a member’ (48%), 9% were almost ready to join, 5% ask GP for advice and only 3% did not know about Coeliac UK. A similar proportion of South Asians (32%) and Caucasians (46%) were members of Coeliac UK, (χ^2^, *p* = 0.09). Of interest, South Asians who were members of Coeliac UK had similar CDAT scores (13 (IQR10-19), *n* = 26) as those who were not members (CDAT 14 (IQR 10-23.5), *n* = 12). Fifty percent of South Asian patients who are members of Coeliac UK and 50% of non-members adhered to the GF diet (< 13 CDAT score). 

A direct logistic regression model was built to explore what practicalities influence patients classified as not adhering to a GF diet by CDAT score > 13, the model contained four independent variables (patient responses to: “My GP does not prescribe GF products”, “I don’t understand the labelling on foods”, “GF foods have an unpleasant taste”, and “whether they were a member of Coeliac UK”). The full model was significant, χ^2^, (4, *n* = 375) = 28.6, *p* < 0.001, and explained 7–10% variance in gluten ingestion (Table 4).

A higher proportion of South Asian patients with CD reported difficulties following the GF diet compared with Caucasians. These difficulties included not understanding what they can eat and a lack of understanding of food labels (Table 3); this is despite all the South Asian patients reporting satisfaction with their appointment with a dietitian. South Asian patients also reported greater dislike for GF foods and viewed them as having a higher expense. In the total cohort there was a higher CDAT score in those whose report disliking GF foods (*p* = 0.028, Table 2), however, CDAT scores were similar in South Asians who liked or disliked GF foods (*p* = 0.9), though only seven South Asians reported liking GF foods.

## 4. Discussion

Adherence to a GF diet is fundamental in the treatment of CD, however a large proportion of Caucasians and South Asians in this study were not adhering to this, a finding which is in agreement with previously published studies [4,6,9]. Determining GF dietary adherence is methodologically challenging [12] and, therefore, we have presented GF dietary adherence as CDAT scores based on a validated questionnaire which correlated well with an evaluation performed by an experienced dietitian [22]. The recent reporting of gluten immunogenic peptides in urine, a novel method to objectively measure gluten ingestion, is likely to help the assessment of GF dietary adherence in the near future [23].

This is the first study to report a significantly higher proportion of patients adhered to a GF diet when receiving GF foods on prescription, compared with those not receiving GF foods on prescription. Hall et al. [4] stated self-reported intentional gluten consumption is higher in those who were not receiving GF foods on prescription (*n* = 40). Robust studies are needed to evaluate the impact of reducing the amount of GF foods prescribed on GF dietary adherence in all population groups. Neither our study nor Hall et al. collected data on the amount of GF food received on prescription nor the reasons why patients were not receiving GF foods on prescription. The cost of GF foods has been shown to be a significant factor in adherence to a GF diet in the U.S. [9,24], therefore, removing prescriptions for GF foods is likely to have financial implications. The majority of patients in our study agreed with the statement ‘GF foods are expensive’, and it is well established that manufactured GF versions of wheat-based foods are substantially more expensive than their gluten-containing versions [15,16]. We did not observe a significant difference in GF dietary adherence scores between those who considered GF foods expense or not. Similarly Leffler et al., [24] highlights that the perception that cost is an important issue in living with CD was not related to GF dietary adherence. However, those who agreed with the statement ‘cost made adherence difficult’ did correlate with poor GF dietary adherence [24]. Our data did not allow us to explore the relative affordability of a GF diet and GF dietary adherence. If patients are not receiving GF foods on prescription, the availability of GF foods to purchase may also impact adherence which to our knowledge has not been investigated to date. Studies have highlighted there is extremely limited GF versions of wheat-based foods in budget supermarkets and local convenience stores [15,16]. This is particularly important when considering the socioeconomic status of patients who are more likely to use these stores [25]. The availability of GF foods is of particular importance where patients are unable to access prescribed GF foods. 

Understanding food labelling is a fundamental skill people with CD need to master to enable them to choose appropriate GF foods. Patients who conveyed an understanding of food labels were more likely to adhere to the GF diet, this is similar to previous studies [20,24,26]. None of the aforementioned studies had an objective measure of understanding of the GF diet, whereas Silvester et al. [3] determined understanding from a GF diet knowledge test, though interestingly the scores did not correlate with self-reported adherence. Assessment of health literacy was overlooked in all studies and could give valuable insight in to the ability of some patients to adhere to a GF diet. Improving understanding of food labels in relation to a GF diet can be achieved through regular contact with a dietitian. Indeed, this could be a factor in the observed greater adherence in those under regular follow-up with healthcare professionals [4].

Research has shown that South Asian Punjabi patients tend to show better satisfaction rates with clinical consultation [27]. Likewise, in our study the satisfaction rate was very good from South Asians, but it would appear that some do have issues with understanding information about a GF diet. South Asians residing in England were significantly more likely to agree with the statement ‘I don’t understand food labelling’ compared with Caucasians. This is particularly concerning as this response was significantly associated with adherence to the GF diet. There is a paucity of research investigating dietary adherence to a GF diet in different ethnic groups, and the number of South Asian responses in our study was relatively small (*n* = 38). It is important to recruit a larger cohort of South Asians with coeliac disease to further our understanding of specific factors influencing their adherence to a GF diet. The only other study we are aware of is over 12 years old whereby Butterworth et al. [20] were unable to identify factors associated with adhering to the GF diet in South Asians (*n* = 21). In other diseases, dietary adherence has been minimally explored e.g., Singh et al., [28] highlighted support from immediate family members helped in the management of diabetes. Our preliminary findings highlight the need for further investigations into ethnic and cultural specific factors which influence GF dietary adherence.

Membership of Coeliac UK was associated with greater GF dietary adherence in our study as has been the case in other countries with coeliac societies [3,4,20,24]. The reasons behind this are likely to be multifactorial. Members are often a self-selected group of patients who may exhibit greater motivation to adhere to the GF diet. In a Canadian patient advocacy group, members had significantly better knowledge of GF foods than non-members [3]. Coeliac societies offer practical advice and support which may contribute to the finding that GF dietary adherence was associated with self-reported ability to follow a GF diet outside the home [3]. Of particular interest from our study was the cohort of South Asian patients where GF dietary adherence was similar in those who were members and those who were not. The reasons behind this are not clear and need to be investigated further but we acknowledge that this may be a reflection on the relatively small number of South Asian participants. 

As with all surveys there were some limitations within our study. The response rate in our survey was lower than the 58% average response rate of data collected from 13 mailed surveys to individuals [29]; however, this obviously depends on the target audience. Furthermore, despite the low adherence rates, there may be some selection bias since the participants who responded may be more motivated and perhaps, therefore, more likely to adhere to a GF diet. Respondents were significantly younger and a greater proportion of Caucasians completed the questionnaire compared with those who did not respond. There is also the possibility of recall bias as some of the data presented is from patients recalling information from their clinical appointments. An additional burden for our patients was a three-day food diary, though not completed in sufficient detail for analysis. Additional factors that may be associated with GF dietary adherence, including psychological traits such as conscientiousness, values, self-efficacy, and perceived tolerance to gluten, and intention to adhere were not assessed within our study [4,5,6,7,22]. It is, thus, understandable that our logistic regression model accounted for only 7–10% of the variance in GF dietary adherence observed. Future research needs to focus on all aspects that influence GF dietary adherence, including the practicalities of adhering to a GF diet, as our study has shown. 

We found that an unacceptably high proportion of patients with coeliac disease were not adhering to a GF diet. We, therefore, recommend retaining adequate GF foods on prescription and improving access to suitably priced GF foods, membership of a coeliac society and regular consultations with a dietitian to provide patients with coeliac disease the knowledge, skills, and support required to adhere to a GF diet.

## Figures and Tables

**Table 1:** Gluten free dietary adherence CDAT scores ^a^ and percentage who self-reported the frequency of consuming gluten.

	Total		Caucasians		South Asians	
	CDAT ^a^	N =	CDAT ^a^	N =	CDAT ^a^	N =
All patients with CD	13 (10–19)	375	13 (10–18)	337	13 (10–19)	38
Females	13 (10–18)	267	13 (10–18)^b^	239	18 (12–21)^b^	27
Males	13 (9–19)	108	15 (9–20)	98	11 (9–13)	10
Frequency of gluten ingestion self-reported by patients with coeliac disease ^c^						
CDAT score < 13	52.2%	197	52.5%	177	52.6%	20
Never	62.4%	234	62.9%	212	57.9%	22
Once a month	25.1%	94	25.5%	86	21.1%	8
Once a week	9.9%	37	9.2%	31	15.8%	6
Daily gluten ingestion	2.1%	8	1.8%	6	5.3%	2

^a^ CDAT (Coeliac Dietary Adherence Test) scores presented as median (interquartile range) values, higher scores indicate poorer adherence to GF diet [22]; ^b^ Mann–Whitney U Test *p* = 0.02; ^c^ Chi-squared analysis indicated no significant difference between groups.

**Table 2:** Adherence to the gluten free diet, CDAT scores ^a^ presented related to responses to statements relating to practical difficulties of adhering to a gluten free (GF) diet.

Responses to the Question: What Difficulties Do You Have in Following the GF Diet?	CDAT Scores ^a^ for Those Who Agree with Statement	N =	CDAT Scores ^a^ for Those Who Disagree with Statement	N =	*p* Value ^b^
I don’t understand what foods I can and cannot eat	16 (10–20)	44	13 (10–18)	331	NS 0.057
I don’t understand the labelling on foods	18 (13–21)	33	13 (9–18)	342	<0.001
I don’t have the time to prepare different meals	15 (11–19)	26	13 (9–19)	349	NS 0.315
GF foods have an unpleasant taste	14 (10–19)	223	12 (9–18)	152	0.028
GF foods are expensive to buy	13 (10–19)	301	14 (9–19)	74	NS 0.77
My GP does not prescribe sufficient amounts of GF products	13 (9–19)	284	13 (10–16)	91	NS 0.25
GP prescribes GF foods	12.5 (9–18)	278	16 (11–16)	97	<0.001
Member of Coeliac UK	12 (9–17)	167	15 (10–20)	208	<0.001

^a^ CDAT (Coeliac Dietary Adherence Test) scores presented median (interquartile range) values, higher scores indicate poorer adherence to GF diet [22]; ^b^ Mann–Whitney U Test. Data collected when guidance to GPs was 18 units. GF; gluten free, NS; not significant.

**Table 3:** Proportion of Caucasians and South Asians who agree with the statements relating to practical difficulties of adhering to a gluten free (GF) diet.

Responses to the Question: What Difficulties Do You Have in Following the GF Diet?	Caucasians		South Asians		Phi	*p* Value ^a^
	%	N = 337	%	N = 38		
I don’t understand what foods I can eat	4.5	15	76.3	29	0.67	< 0.001
I don’t understand food labelling	3.9	13	52.6	20	0.52	< 0.001
I don’t have time to prepare the different meals	7.4	25	2.6	1	NS	0.27
GF foods are unpleasant	57.0	192	81.6	31	0.15	0.003
GF foods are expensive	78.3	264	97.4	37	0.14	0.005
My GP does not prescribe enough GF foods	73.6	248	94.7	36	0.15	0.004
GP prescribes GF foods	72.4	244	89.5	34	−0.12	0.02
Member of Coeliac UK	46.0	155	31.6	12	NS	0.09

^a^ Chi squared, GF; gluten free, NS = non-significant.

**Table 4:** The practicalities significantly impacting upon adhering to a gluten-free (GF) diet.

	Odds Ratio (OR)	95% CI (Lower)	Upper	*p* Value
I don’t understand the labelling on foods	2.56	1.31	3.47	0.024
My GP does not prescribe GF products	2.14	1.31	3.47	0.002
Non-member of Coeliac UK	1.72	1.12	2.64	0.013
GF foods have an unpleasant taste	1.43	0.93	2.20	0.107

Logistic regression model: χ^2^, (4, *n* = 375) = 28.6, *p* < 0.001, the model explained 7–10% variance in gluten ingestion. CDAT score < 13 represents GF dietary adherence [9].

## Supplementary Materials

The following are available online at www.mdpi.com/2072-6643/9/7/705/s1.
