# Supplementary material for: Adherence to a Gluten Free Diet Is Associated with Receiving Gluten Free Foods on Prescription and Understanding Food Labelling
Source: Nutrients. 2017 Jul 6;9(7):705. doi: 10.3390/nu9070705 (PMC5537820; doi:10.3390/nu9070705)
Supplement: Supplementary file 1 [file nutrients-09-00705-s001.zip › nutrients-202561-supplementary.pdf]

Questionnaires:

Instruction for filling the form: (Please read these carefully before you complete the form)

1. Please circle or tick the relevant option(s)
2. Where appropriate you may select more than one option
3. Please do not leave any question blank
4. If you do not know the answer, please write “don’t know” or “DK”
5. Please post the questionnaire back to us in the envelope provided.

Part A:

1. Sex Male Female

2. Age

18–30 31–40 41–50 51–60 more than 60

3. Which ethnic group do you belong too?

European Muslim Sikh Hindu

4. Are you a vegetarian?

Yes No

5. At what age were you diagnosed by the hospital to be suffering from coeliac disease?

18–30 31–40 41–50 51–60 more than 60

6. Did you have health problems in childhood?

Yes No

7. What prompted you to consult your General Practitioner (GP)? [Please tick relevant option(s)]

Fatigue/ Hair loss

Stomach pains/ bloating / Skin rash

Poor appetite/ Diarrhoea

Nausea/Vomiting /Mouth ulceration

Weight loss /Family history

Joint pains /Difficulty walking

University of Roehampton London

8. How long had you been experiencing symptoms before you were diagnosed?

Less than 6 months   6 months to 1 year   1 year to 3 years   more than 3 years

9. Did you know what coeliac disease was before you were diagnosed?

Yes   No

10. At the time of your diagnosis, what was discussed at your consultation with your hospital doctor? [Please tick relevant option(s)]

Explained what coeliac disease was   Told me to follow a strict gluten-free diet

Referred me to a dietician   Arranged a follow-up appointment

Gave written information   Other (please state)

11. Were you satisfied with the information given?

Yes   No

12. If you were referred to a dietician, what advice were you given? [Please tick relevant option(s)]

Explained the diagnosis and the reasons for the diet

Discussed a gluten-free diet

Discussed the Coeliac Society and local groups

Provided an information pack (containing diet sheet, food list, starter packs etc)

Discussed the prescribing of gluten-free products

A follow-up appointment was made

Given a contact telephone number for advice, if needed

13. Were you satisfied with the information given?

Yes No

If "No", please state why:

14. Do you think the dietician should play an important role in the long-term management of Coeliac Disease?

Yes No

15. How often do you include gluten containing foods in your diet?

Never Once a month Once a week Daily Other please specify

16. What difficulties do you have in following a gluten-free diet? [Please tick relevant option(s)]

I don't understand what foods I can and cannot eat

I don't have the time to prepare different meals

Gluten-free foods have an unpleasant taste

Gluten-free foods are expensive to buy

My GP does not prescribe sufficient amounts of gluten-free products

I don't feel any different on a gluten-free diet

I don't understand the labelling on foods

17. Do you have symptoms when you eat food containing gluten?

Yes No

If yes, what symptoms do you experience? [Please tick relevant option(s)]

Nausea/vomiting Stomach pains

Diarrhoea Fatigue/tiredness

Mouth ulcers

Others (please specify)

18. Do you get gluten-free products on prescription?

Yes

No

If No, did you know that they are available on prescription?

Yes

No

If yes, do you get sufficient amounts of gluten-free products on prescription?

Yes

No

19. Are you a member of the Coeliac Society?

Yes (please go to question 20)

No

If you are not a member, is it because:- [please tick relevant option(s)]

You didn't know about it

You have a contact address, but haven't joined yet

You don't feel it is important

If I need advice, I will ask my GP

Other (please specify)

Part B:

Please circle one option for each question in the columns from 1 to 5.

Questions	1	2	3	4	5
Have you been bothered by low energy level during the past 4 weeks?	None of the time	A little of the time	Some of the time	Most of the time	All of the time
Have you been bothered by headaches during the past 4 weeks?	None of the time	A little of the time	Some of the time	Most of the time	All of the time
I am able to follow a GFD when dining outside my home	Strongly agree	Somewhat agree	Neither agree nor disagree	Somewhat disagree	Strongly disagree
Before I do something I carefully consider the consequences	Strongly agree	Somewhat agree	Neither agree nor disagree	Somewhat disagree	Strongly disagree
I do not consider myself a failure	Strongly agree	Somewhat agree	Neither agree nor disagree	Somewhat disagree	Strongly disagree
How important to your health are accidental gluten exposures?	Very important	Somewhat important	Neutral/unsure	A little important	Not at all important
Over the past 4 weeks, how many times have you eaten foods containing gluten on purpose?	0-never	1-2	3-5	6-10	>10

Thank you for completing the questionnaire. Please use the enclosed stamped addressed envelope to return it to us.
